# Analysis of bacteria-challenged wild silkmoth, *Antheraea mylitta *(lepidoptera) transcriptome reveals potential immune genes

**DOI:** 10.1186/1471-2164-7-184

**Published:** 2006-07-21

**Authors:** Archana S Gandhe, KP Arunkumar, Serene H John, J Nagaraju

**Affiliations:** 1 Laboratory of Molecular Genetics, Centre for DNA Fingerprinting and Diagnostics, ECIL Road, Nacharam, Hyderabad 500076, India

## Abstract

**Background:**

In the recent years a strong resemblance has been observed between the insect immune system and the mammalian innate immune mechanisms suggesting their common origin. Among the insects, only the dipterans (*Drosophila *and various mosquito species) have been widely investigated for their immune responses towards diverse pathogens. In the present study we constructed and analysed the immune transcriptome of the lepidopteran *Antheraea mylitta*, an economically important Indian tasar silkmoth with a view to unravel the potential immune-related genes and pathways.

**Results:**

An expressed sequence tag (EST) library was constructed from mRNA obtained from fat bodies of *A. mylitta *larvae that had been challenged by infection with *Escherichia coli *cells. We identified 719 unique ESTs from a total of 1412 sequences so generated. A third of the transcriptome showed similarity with previously characterized immune-related genes that included both the known and putative immune genes. Of the four putative novel defence proteins (DFPs) annotated by PSI-BLAST three showed similarity to extracellular matrix proteins from vertebrates implicated in innate immunity, while the fourth was similar to, yet distinct from, the anti-microbial protein cecropin. Finally, we analysed the expression profiles of 15 potential immune-related genes, and the majority of them were induced more prominently with *E. coli *compared to *Micrococcus luteus*. We also identified several unknown proteins, some of which could have probable immune-related functions based on the results of the ProDom analysis.

**Conclusion:**

The present study has identified many potential immune-related genes in *A. mylitta *some of which are vertebrate homologues and others are hitherto unreported putative defence proteins. Several genes were present as members of gene families, as has also been observed in other insect species.

## Background

Insects are evolutionarily successful organisms and occupy almost all habitats in nature. An efficient immune system is one of the attributes for this evolutionary success. However, unlike mammals, the insects lack an adaptive immune system. The insect immune response is comprised of cellular and humoral components. The former involves the action of haemocytes in phagocytosis of microbes, encapsulation of large pathogens and nodule formation [1] whereas the latter involves activation of prophenoloxidase cascade leading to melanisation of invading microorganisms [2] and synthesis of a battery of anti-microbial peptides [3].

Insect immunity is well studied in dipterans such as fruit flies and mosquito species [4-7]. Only limited information is available on genes induced on pathogen challenge in a few lepidopteran species that include the domesticated silkmoth, *Bombyx mori *[8], Cecropia moth,*Hyalophora cecropia *[9] and tobacco hornworm,*Manduca sexta *[10] and in these too the immune response pathways employed to combat pathogen infections remain to be fully characterised.

Abundant genetic resources are now available for *B. mori*, with a 9 X shotgun sequence coverage of its genome and more than 100,000 ESTs in dbEST (NCBI) [11-13]. With reference to insect immunity, the ESTs have been obtained from baculovirus-infected *B. mori *cultured cells and pupae, but no large scale information on bacteria-induced immune genes is as yet available.

In this study, we have constructed and analysed an immune transcriptome following bacterial challenge of the Indian tasar wild silkmoth, *Antheraea mylitta*, an economically important lepidopteran cultivated for silk production. Prior information on immune response genes in wild silkmoths is lacking except for a few peripheral studies. Two proteins from *A. mylitta *– a lysozyme protein, 3-D structure of which is elucidated [14] and a protease inhibitor have been characterized [15]. We chose to examine the fat body transcriptome since it is a major immune organ in insects, analogous to the mammalian liver. We generated a total of 1412 ESTs, of which 31% could be ascribed to putative immune functions. We also validated the upregulation of a selected subset of genes from the immune transcriptome by semi-quantitative RT-PCR.

## Results and discussion

**Figure 1:**
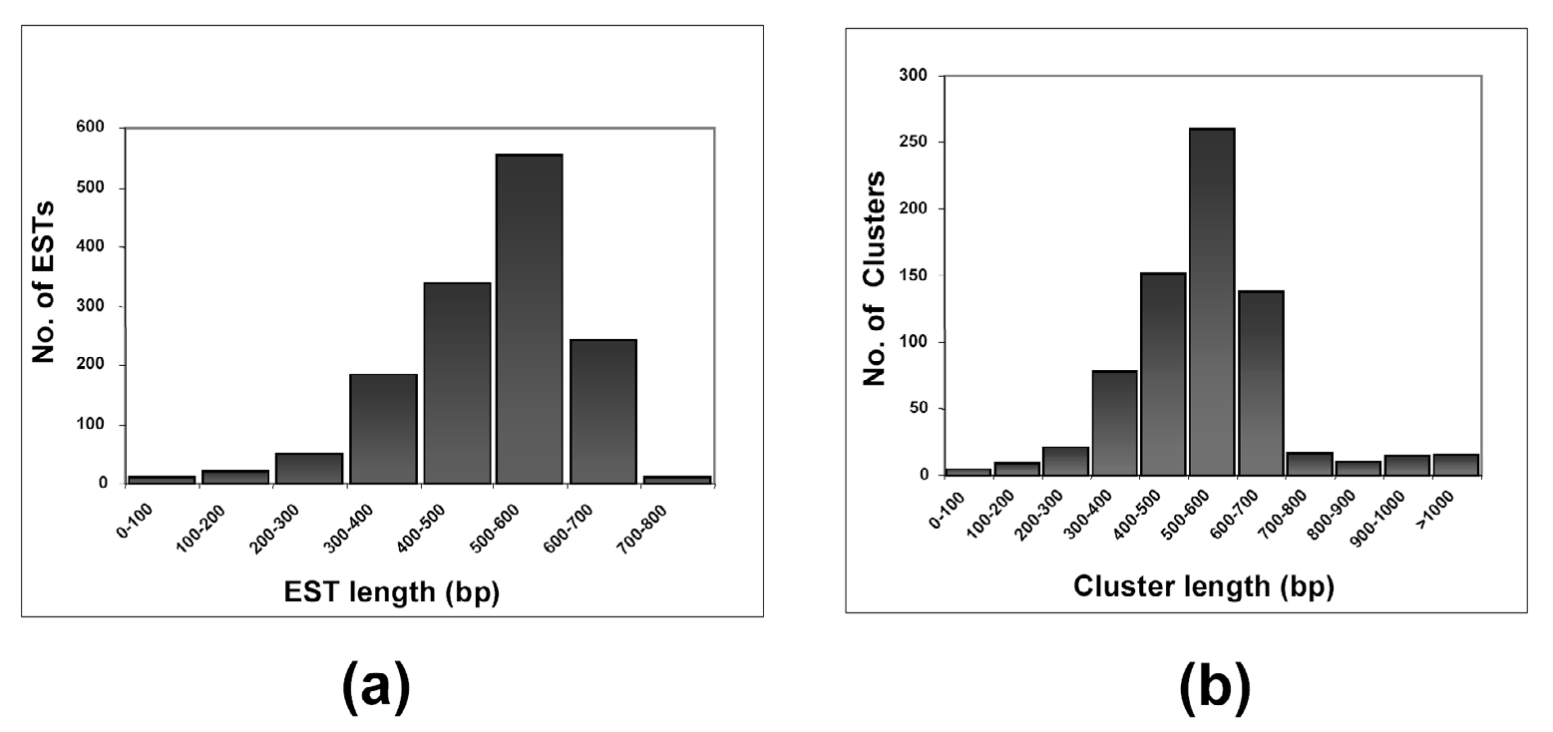
Distribution of read lengths of *A. mylitta *ESTs and clusters. (a) Read lengths of 1412 ESTs. (b) Read lengths of clusters.

As described in Materials and Methods, we constructed a cDNA library from fat body tissues of *E. coli*- challenged *A. mylitta *larvae and randomly sequenced a large number of inserts from the library. By running TGICL program, we obtained 719 clusters from a total of 1412 ESTs, of which 166 were contigs (comprising 859 ESTs) and 553 were singletons. The majority of the EST clusters were 500 to 600 bp, with an average of 524 bp and a maximum of 1994 bp (Figure 1). Each of these clusters potentially represents a unique gene from *A. mylitta*, and our results have hence expanded the number of genes known for this organism from the handful previously known genes. The 1412 EST sequences (accession numbers EB742119- EB743530) can be accessed at the NCBI EST sequence database, dbEST and the 719 clusters can be accessed at URL mentioned in references [16].

### BLAST analysis

**Figure 2:**
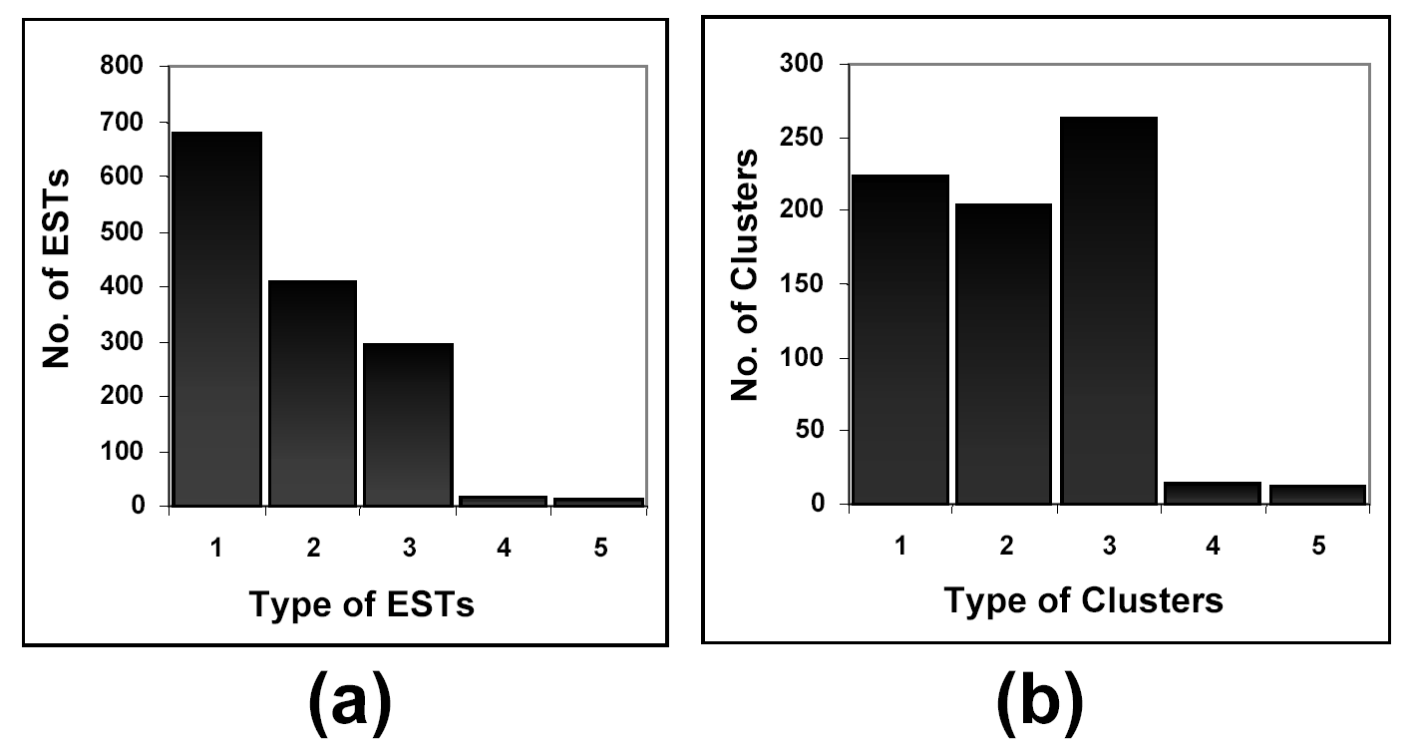
Classification of ESTs (a) and Clusters (b) based on the number of sequences showing similarity to insect and or mammalian proteins in NCBI protein nr database. X-axis: 1) Insect, 2) Insect and Mammal, 3) Unknown, 4) Mammal, 5) Neither Insect nor Mammal, Y-axis: Number of sequences.

The different ESTs were classified into categories such as immune-related, housekeeping, hypothetical insect proteins, hypothetical non-insect proteins based on the homology in NCBI protein BLAST (Table 1). Of the 1412 ESTs, 432 (31%) showed similarity to known or putative insect immune proteins; thus, even though the cDNA library was not normalized, a significant proportion (one-third) of the transcriptome was represented by putative immune-related genes. A total of 569 ESTs (39%) were homologous to housekeeping genes and proteins involved in functions other than immunity. Functional class distribution of the ESTs is depicted in Table 1. Based on the sequence similarity, 679 of the 1412 ESTs were classified as insect-specific (224 of the 719 clusters) and 409 ESTs (204 clusters) were common to both insects and mammals (Figure 2a and 2b). The number of ESTs showing similarity to only the mammalian proteins was 14 and those showing similarity neither to insects nor to mammals were as few as 13. Gene Ontology analysis of the EST sequences was also carried out and the details are provided as supplementary data (see Additional data file 1).

### Domain search

The sequences, which could not be assigned any function based on homology search in NCBI, were searched for conserved domains in ProDom database. Of the 260 clusters that had no matches with known proteins in NCBI BLAST, we could assign protein domain families to 196 clusters based on the ProDom search (see Additional data file 2). The remaining sequences did not show any hits in ProDom and should be further analysed by other specialized computational tools.

### Signal peptide and transmembrane domains

We further screened the unannotated proteins (no hits or hits with hypothetical proteins in NCBI database) for the presence of signal peptide and absence of transmembrane domains. This characteristic of many of the immune proteins has been utilized to screen probable immune-related genes in large-scale transcriptome studies [17], although the reliability of this criterion to identify the immune related genes remains to be experimentally tested. In the present study, 25 out of 260 genes tested fulfilled these criteria and could be considered as potential immune-responsive genes. However, since all the 260 clusters checked are not all full-length sequences, it is possible that we may have missed some others amongst them that represent gene products harbouring signal peptide without transmembrane domain, and hence the actual number may be higher.

### Putative immune proteins

**Figure 3:**
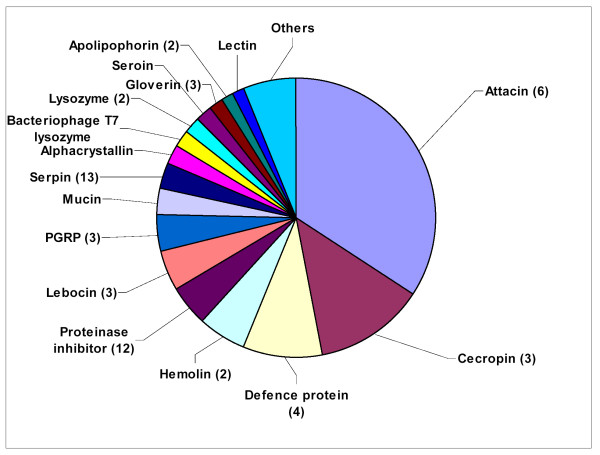
Distribution of immune-related transcripts in *A. mylitta *fat body transcriptome. The figure in parenthesis indicates the number of isoforms identified in that particular gene family.

A total of 80 clusters were assigned a putative immune function based on their similarity with previously characterized immune response genes and their distribution pattern is shown in Figure 3. These putative immune proteins were categorized into different functional groups – i. anti-microbial proteins, ii. pattern recognition receptors (PRRs), iii. proteases and protease inhibitors and iv. putative defence proteins with unknown function. A subset of these putative immune genes (thirty-eight) is represented with the details like GenBank accession numbers, putative function, E-value (BLAST) etc (see Additional data file 3). Homologues of several other proteins, implicated in immunity such as antioxidants, ferritins, transferrins, apolipophorins and apoptotic pathway components were also found, but are not further described here.

#### Putative anti-microbial proteins

Attacin-like proteins were the most abundantly expressed transcripts in terms of EST copy number in the immune transcriptome (Table 2). Six different types of putative attacins accounted for 34% (148 ESTs) of the immune transcripts. A previous study had reported the presence of four types of attacins in *D. melanogaster *[18] and a similar study in immunized pupae of *H. cecropia *led to the isolation of six closely related types of attacins [9]. Second in the list of highly expressed genes were cecropin-like proteins (55 ESTs, ~13%). Three types of cecropin homologues were found in the *A. mylitta *transcriptome and a previous study in *H. cecropia *had similarly shown the existence of three types of cecropins [19]. Three types of putative lysozyme transcripts were detected, one of which was homologous to a bacteriophage T7 lysozyme-like protein reported in *B. mori *that was later named, based on functional analysis, as a peptidoglycan recognition protein [20]. The second was homologous to the bacteriolytic lepidopteran lysozymes known to lyse the bacterial cell wall [21]. The third was a lysozyme-like protein that lacks one or both of catalytic residues essential for muramidase activity. This protein has not been reported in lepidopterans before and is homologous to dipteran lysozyme-like proteins of unknown function. Three types of putative lebocins and gloverins, which are the lepidopteran specific anti-bacterial genes, were also observed in *A. mylitta *transcriptome.

The data generated in the present study conformed to the trend of anti-microbial genes being present as multiple gene families as observed in many other insect species and highlighted the essentiality of these genes in the organism [22]. Another interesting finding with respect to immune response was the presence of a protein identical to seroin. This protein is reported to express in the silk glands of *B. mori *and is known to protect the cocoon from microbes [23]. A homologue of anti-fungal protein, gallerimycin, previously characterized from *Galleria mellonella *and shown to be induced by LPS injection [24], was also found.

#### Putative pattern recognition receptors (PRRs)

PRRs in the insects bind to and detect pathogen associated molecular patterns (PAMPs) like lipopolysaccharide, peptidoglycan, β 1–3 glucan, lipotechoic acid etc [9,25]. The putative PRRs identified in the present immune transcriptome analysis were hemolins, peptidoglycan recognition proteins (PGRPs), gram-negative binding proteins (GNBPs), lectins and mucins. *A. mylitta *fat body transcriptome revealed two different proteins resembling hemolin, an immune inducible protein implicated in insect immunity [26] and it would be worthwhile to study the function and specificities of the different proteins of hemolin family. Three types of PGRP-like proteins were found in the *A. mylitta *immune repertoire in the present study as compared to *D. melanogaster *where 13 PGRP genes are known to be involved in the activation of the various effector pathways in immunity [27].

#### Putative proteases and protease inhibitors

Various proteases and protease inhibitors regulate the diverse immune mechanisms like melanization, phagocytosis and induction of anti-microbial peptides [10]. A homologue of a prophenoloxidase activating protease characterized in *B. mori *and *M. sexta *[11] was also found in the *A. mylitta *immune transcriptome. Several other classes of putative proteases like cysteine proteases, serine proteases and metallo-proteases were also identified. As many as thirteen distinct serpin-like (serine protease inhibitors) and twelve potential protease inhibitors were detected in the immune transcriptome. Five different serpins have earlier been identified in *M. sexta *and shown to differ in the induction pattern upon immune challenge [1]. In the light of these studies, the information on serpins and protease inhibitors from the *A. mylitta *immune transcriptome will prove to be invaluable in further understanding of various immune pathways in insects.

#### Putative defence proteins with unknown function

Several potential new members of known protein families were identified in the present study. Among the new members, one was a putative lysozyme-like protein described in the previous section. An array of putative proteinase inhibitors and proteases were also found. Many of them are new, and their study would enhance our understanding of the mechanisms of proteolytic cascades in insect innate immunity. A few immunoglobulin (Ig) like molecules were identified by ProDom search (see Additional data file 2). Ig-like molecules- hemolin [26] and more recently Dscam have been implicated in insect immunity [28,29] and it would be interesting to evaluate the role of these putative Ig-like molecules in insects. Among the potential immune proteins, we describe below in more detail four putative defence proteins (DFPs), for two of which (DFP-1 and DFP-4) we have confirmed the induction upon *E. coli *infection by semi-quantitative RT-PCR.

#### DFP-1, DFP-2 and DFP-3

**Figure 4:**
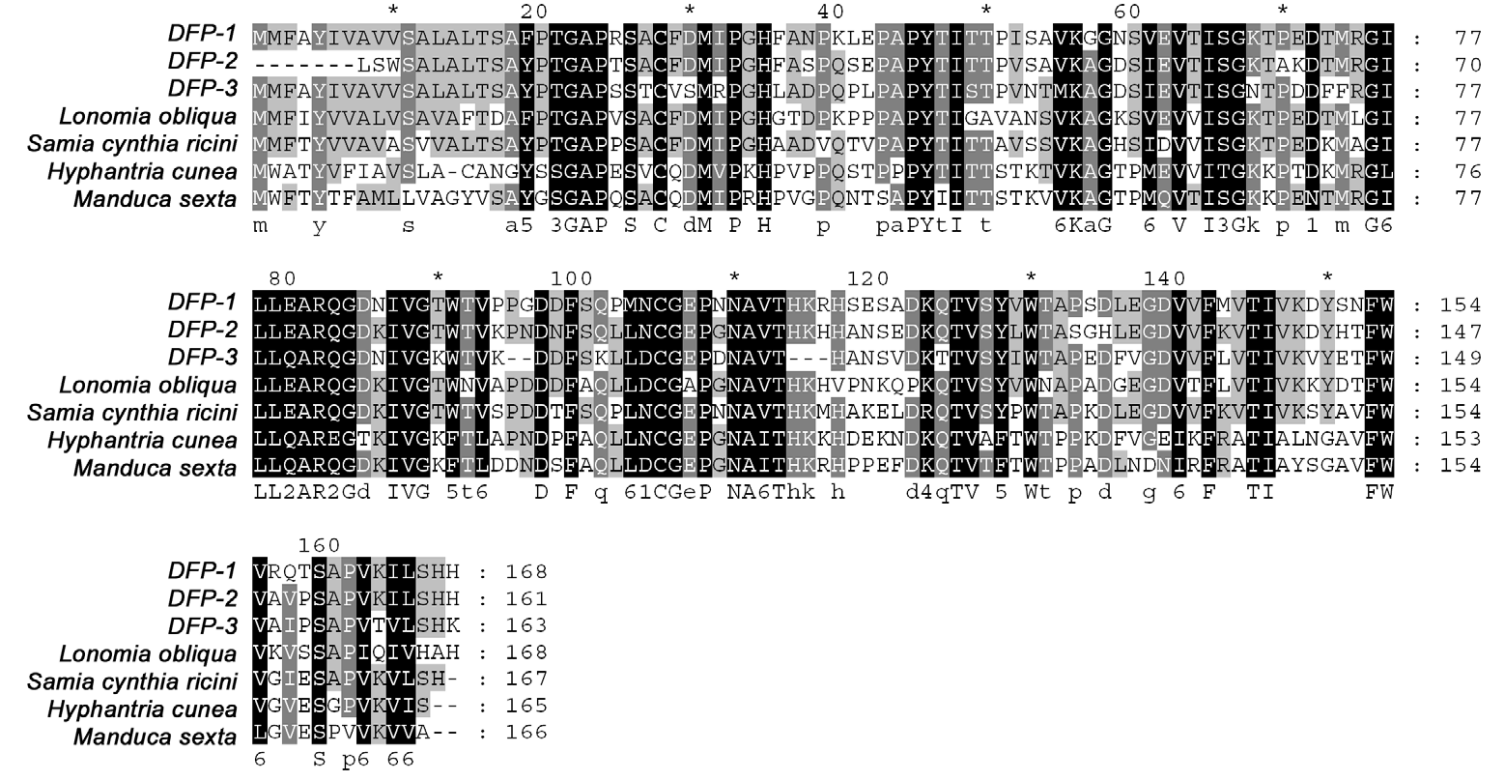
Multiple sequence alignment of DFP-1, 2 and 3 amino acid sequences with homologues from other lepidopterans. *Lonomia obliqua *[GenBank:AAV91350], *Manduca sexta *[GenBank:AAO21507], *Samia cynthia ricini *[GenBank:BAD05929], *Hyphantria cunea *[GenBank:AAD09280] protein sequences, respectively. Black and grey shades indicate the identical and similar amino acid residues, respectively.

We have grouped these three proteins together, as they are 70–85% similar to each other (DFP-1&DFP-3 = 85%, DFP-1&DFP-2 = 79% and DFP-2&DFP-3 = 77%). All of them have a signal peptide and appear to be secretory proteins. DFP-1 was abundantly expressed in the immune transcriptome suggesting its possible involvement in immunity. In addition, these three proteins showed high similarity to immune induced unknown proteins from other lepidopterans like *Hyphantria cunea*, *Samia cynthia ricini*,*M. sexta *and *Lonomia obliqua *[10,30-32] (Figure 4), as also with some hypothetical proteins from other insects and vertebrates in the NCBI database (see Additional data file 4). Analysis by Position-specific iterative BLAST (PSI-BLAST) [33] revealed similarity of DFP-1, 2 and 3 to the vertebrate extracellular matrix proteins (ECM), stromal cell derived factor receptor-2, spondin and reelin and possessed the common domain termed as 'reeler' (Table 3). These ECM proteins are involved in the central nervous system signaling and immune mechanisms like 'signaling' and 'pathogen recognition' [34,35]. Stromal cell derived factor/receptor complex has been shown to activate JAK/STAT pathway and mediate the migration and proliferation of haematopoietic cells [35]. Recently, mindin a protein belonging to the F-spondin family has been shown to act as a pathogen recognition receptor in mice [34]. Also, spondin has been shown to be upregulated in *Drosophila *upon bacterial infection by microarray analysis [36]. The similarity of DFP-1, 2 and 3 to molecules involved in immune responses in vertebrates further support the immune-related role of these new proteins.

#### DFP-4

**Figure 5:**
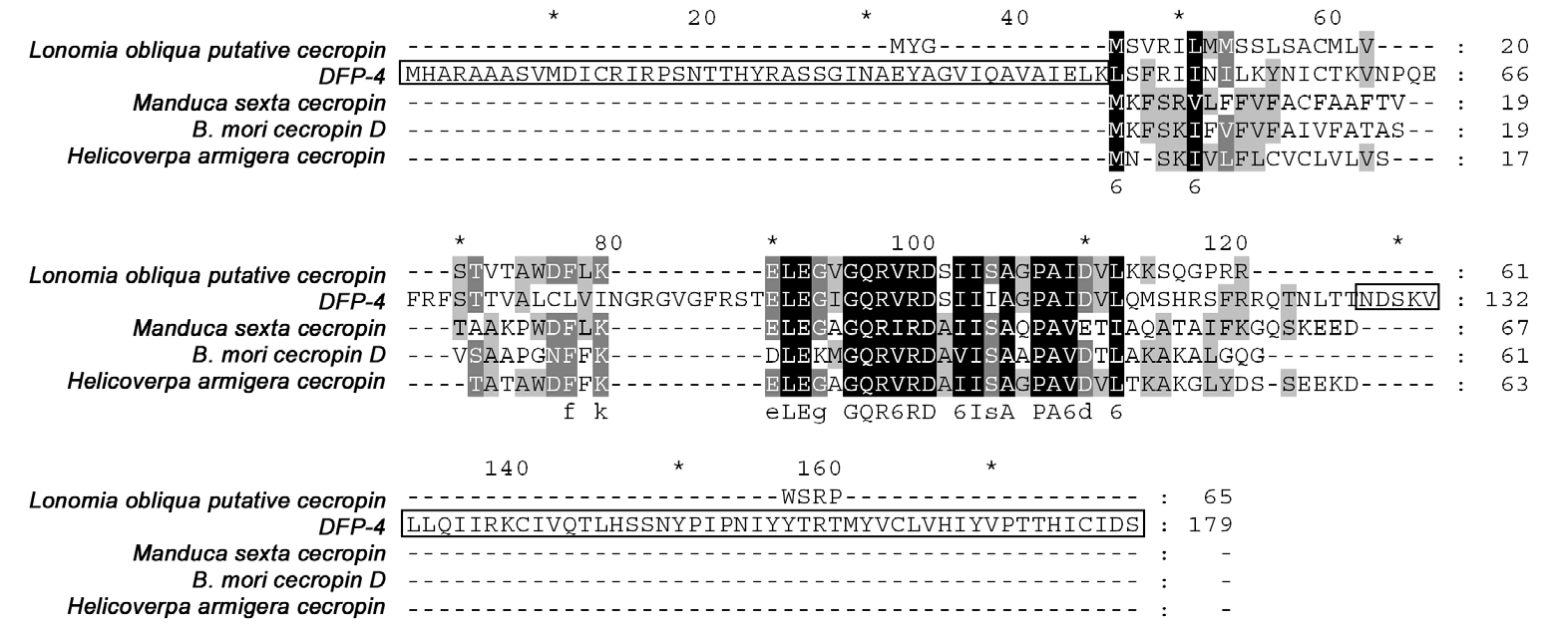
Multiple sequence alignment of DFP-4 with cecropins from other lepidopterans. *L. obliqua *putative cecropin [GenBank:AAV91462], *M. sexta *cecropin [GenBank:AAO74638], *B. mori *cecropin D [GenBank:BAE53371], *Helicoverpa armigera *cecropin [GenBank:AAX51193.1]. The unique regions of DFP-4 (no homology to cecropins) are boxed.

This protein was particularly intriguing as it showed similarity to cecropin, the insect anti-bacterial peptides in the primary sequence BLAST analysis. The multiple alignment of the various cecropins and DFP-4 is shown in Figure 5. Based on the SignalP prediction, DFP-4 is likely to be a non-secretory intracellular protein, unlike cecropins that are secreted into the haemolymph. Cecropins are small 5–6 KD peptides whereas DFP-4 is a 17 KD protein with additional unrelated regions at the N and C-termini. The exact role of DFP-4 is not clear and needs to be investigated.

### Expression profile

**Figure 6:**
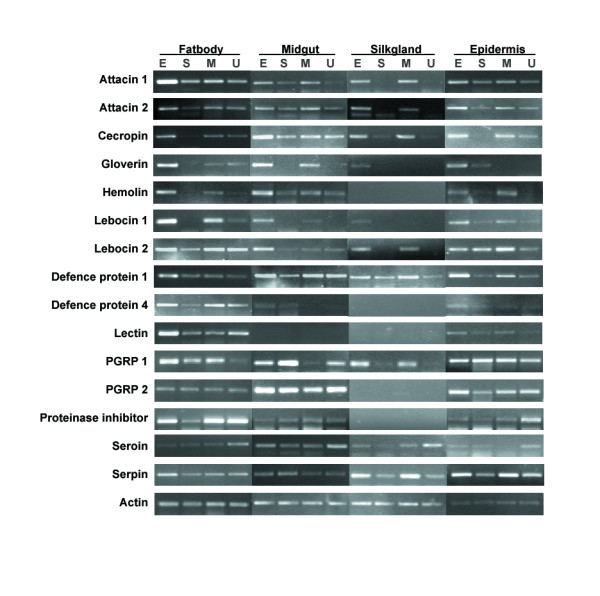
RT-PCR profiles of 15 putative immune genes from 4 differentially challenged tissues of 5^th ^instar larvae. E- *E. coli *challenged, M- *M. luteus *challenged, S- saline-injected, U- unchallenged. Actin was used as an endogenous control. The GenBank accession numbers of the 15 genes are provided as additional data (see Additional data file 5).

The transcriptome under study most likely represents a plethora of *E. coli*-induced genes in the fat body of the tasar silkmoth. We validated 15 putative immune response genes by semi-quantitative RT-PCR, and their expression profiles are shown in Figure 6. All but two genes, a putative protease inhibitor [GenBank:DQ666519] and a seroin gene [GenBank:DQ666525] were upregulated upon infection. The two DFPs tested, DFP-1 [GenBank:DQ666501] and DFP-4 [GenBank:DQ666503], were induced by both *E. coli *and *M. luteus*. DFP-1 was highly expressed in all the tissues with a more prominent expression in mid gut whereas DFP-4 was exclusively expressed in fat body (Figure 6). We analysed the expression pattern of the aforementioned genes in larval tissues differentially challenged with *E. coli *(gram-negative) or *M. luteus *(gram-positive) and compared with challenged or mock-challenged (saline-injected) tissues as negative controls. Most of the genes analysed were expressed more prominently upon infection with *E. coli *than with *M. luteus *suggesting that there may be differential responses towards different pathogens. Two pathways of differential immune induction have been identified in *Drosophila*. Gram-positive bacteria and fungi induce the Toll pathway whereas gram-negative bacteria evoke the Imd pathway [37], but the degree of conservation of these pathways between Drosophila and lepidopterans is not known.

## Conclusion

The immune response in insects is dynamic and different effector genes are likely expressed at different time points during infection, contributing to the ability of the insects to ward off infections in spite of the absence of adaptive immunity. The current transcriptome represents genes likely expressed upon *E. coli *infection in the *A. mylitta *fat body at 24 hrs post infection. Unexpectedly, the Imd pathway components that are implicated in the activation of various effector pathways upon gram-negative bacterial infection in *Drosophila *were not present in our transcriptome. The 24-hour post infection period may have been non-optimal for expression of some genes, and since the EST library was not normalized the less abundant transcripts may have gone undetected. Alternatively, it is possible that other pathways are involved in the immune induction in the moths.

The present study has increased the repertoire of lepidopteran-specific putative immune response genes by several hundred-fold. This will be a valuable resource for lepidopteran-specific immune studies in particular and insect immune studies in general.

## Methods

### Insects, bacterial inoculation and tissue collection

*A. mylitta*, 5^th ^instar, day 3 larvae were procured from Regional Research Station, Warangal, Andhra Pradesh. Log phase *E. coli *cells (DH5α), washed and resuspended in saline (0.3 M NaCl, 0.005 M KCl), were injected into the haemocoel of the larvae as described earlier [38]. At 24 hours post infection (hpi), larvae were dissected to isolate fat body, and the tissue was flash frozen in liquid N_2 _and then stored at -70°C till further use.

### cDNA library construction and generation of ESTs

Total RNA was extracted from the fat body using Trizol reagent (Invitrogen). The complementary DNA synthesis was carried out using Stratagene ZAP-cDNA^® ^synthesis kit following manufacturer's instructions. Directional cDNA library was constructed by cloning of cDNA fragments into pBluescript II SK (+) vector and electroporation into *E. coli *strain DH10B. Insert-containing plasmid clones were sequenced with RV-M primer (5'GAG CGG ATA ACA ATT TCA CAC AGG 3') with the aid of MegaBACE3000 sequencer.

### EST processing

Raw sequences obtained from sequence chromatograms were processed using several programs. A cut-off Phred Quality Value of ≥15 was assigned to extract quality sequences from chromatograms. The quality sequences were screened for the presence of vector sequences using 'Cross Match' program [39]. Then masked vector sequences were automatically removed by in-house developed trimming tool. Sequences shorter than 50 bases were removed. The resulting high-quality sequences were assembled into sequence contigs with the TGICL program [40], which initially makes clusters using MegaBLAST and thereafter makes an assembly using CAP3 for each cluster generated in the first step. A cluster is defined as a unique sequence obtained either by multiple alignment of many sequences that are > 95% similar or derived from a single sequence. A cluster containing ≥2 ESTs is termed a contig and that containing only one sequence, a singleton.

The unique putative gene sequences obtained by clustering and assembly were annotated by running BLAST [41] against non-redundant (*nr*) protein database of NCBI. Further, BLAST output was parsed to classify the putative gene transcripts into different functional classes.

### Analysis of unknown proteins

Proteins that did not show any significant hits in NCBI *nr *database or showed similarity to unknown or hypothetical proteins were characterized by additional computational tools.

#### a. Domain search

For the sequences showing high similarity to hypothetical and/or unknown proteins, and those showing weak similarity to *nr *protein database, domain search was performed using ProDom [42]. Putative function was assigned based on the type of domains found.

#### b. Signal peptide and transmembrane domain analysis

Presence of transmembrane domains and signal peptide analysis was done on the transcripts not showing any significant hits in NCBI database. The signal peptide analysis was done by SignalP software [43] and trans-membrane domain analysis was done with TMHMM program [44].

#### c. Functional annotation with PSI-BLAST

The functional annotation of four novel immune upregulated transcripts (DFPs) was done using PSI-BLAST [33].

### Expression profile

*A. mylitta *larvae were differentially challenged- a) Unchallenged, b) Saline-injected, c) *M. luteus *and d) *E. coli*. Log phase *E. coli *and *M. luteus *bacteria, washed and resuspended in insect saline (0.3 M NaCl, 0.005 M KCl) were injected (30 μl) into a set of *A. mylitta *larvae. One set each of saline-injected and uninjected larvae were kept as a control. Four tissues, namely fat body, epidermis, mid gut, and silk gland were dissected out and flash frozen in liquid nitrogen. Total RNA was isolated using Trizol reagent. To remove genomic DNA contamination, total RNA was treated with RNAse free DNAse (NEB) as prescribed by the manufacturers. cDNA was synthesized using MMLV reverse transcriptase (Invitrogen) and oligo dT primers from 1 μg of total RNA. The primers were designed for the selected ESTs by Primer3 software [45].

Semi-quantitative RT-PCR was carried out for all the four differentially challenged tissues using an Eppendorf master cycler under the following conditions- 94°C, 2 min- initial denaturation, 27 cycles (94°C – 30 s, 58°C- 30s, 72°C-2 mins) and a final elongation at 72°C for 10 mins. Actin cDNA was amplified as an endogenous control. PCR reaction components included: 1X buffer, 100μM dNTPs, 1.5 mM MgCl2, 0.5 units Taq polymerase (MBI), 0.5 μM primers. Primer sequences are enlisted in additional data (see Additional data file 5).

### Obtaining full-length cDNA by 5' RACE

We had obtained full-length coding sequences of the DFP-1, 2 and 3 through EST sequencing. To acquire full-length DFP-4 cDNA, we carried out 5' RACE PCR using the 5' RACE kit (Clontech). The 5' ends were amplified by using an adaptor primer and a reverse gene specific primer. PCR was performed for 25 cycles in an Eppendorf master cycler. A 300 bp band was isolated, sequenced and confirmed to be the 5' DFP-4 sequences.

## Authors' contributions

AG and AKP conceived and designed the study, participated in the bioinformatics and validation analysis, and drafted the manuscript. SH carried out the expression profile analysis, assisted in the bioinformatics analysis and manuscript preparation. JN conceptualised the project, critically reviewed the manuscript and provided guidance. All the authors read and approved the final manuscript.

## Supplementary Material

Additional data file 1 Additional data file 1 includes the results, methodology and schematic representation of Gene Ontology analysis of the ESTs. Click here for file

Additional data file 2 Additional data file 2 is a table listing the ProDom based annotation of the clusters that did not find any matches in NCBI protein BLAST analysis. Click here for file

Additional data file 3 A table representing some of the putative immune genes from the transcriptome is provided as an Additional data file 3 Click here for file

Additional data file 4 Additional data file 4 is a table that lists the hits obtained by NCBI protein BLAST analysis of DFP-1, 2, 3 and 4. Click here for file

Additional file 5 Primer sequences of the 15 transcripts analysed by RT-PCR have been provided in Additional data file 5. Click here for file

## Figures and Tables

**Table 1:** Classification of EST functional class categories based on similarity searches with NCBI protein database.

**Category**	**No. of ESTs**
Total no. of ESTs	1412 (100%)
Immune-related	432 (31%)
Housekeeping genes and other genes not involved in immunity	569 (39%)
Hypothetical/unknown insect proteins	135 (9.5%)
Hypothetical/unknown non-insect proteins	50 (3.5%)
Weak homology in NCBI*	170 (12%)
No homology in NCBI	56 (3.9%)

Percentage value of each category is in parenthesis. *- Hits with relatively less similarity (E value>1e – 05).

**Table 2:** The abundant genes in *A. mylitta *transcriptome.

**Gene definition**	**No of ESTs**
Attacin	96
Cecropin D	31
Defense protein 1	30
Ribosomal protein S2, Attacin	26
Elongation factor 1α	24
Hemolin	23
Attacin	22
Cecropin	17
Ferritin	15
Mucin	13

The number of ESTs indicates the transcript abundance for each of the category

**Table 3:** Proteins similar to putative defence proteins (DFPs) revealed by PSI-BLAST analysis.

**Novel proteins from *A. mylitta***	**Description and function of proteins similar to DFPs.**
**DFP-1, DFP-2, DFP-3**	**1. Stromal cell derived factor receptor 2 homologue** *Homo sapiens *[GenBank:NM_001013660.1] (catecholamine catabolism) reeler domain
	
	**2. Spondin** *Bos taurus *[GenBank:Q9GLX9] *H. sapiens *[GenBank:Q9HCB6] Extracellular matrix, cell adhesion protein that promotes the attachment of spinal cord and sensory neuron cells
	
	**3. Reelin precursor** *H. sapiens *[GenBank:P78509] Extracellular matrix serine protease. Enzymatic activity is important for the modulation of cell adhesion

**DFP-4**	**Cecropin D precursor** *L. obliqua *[GenBank:AAV91462]

The GenBank accession numbers are given in parentheses.
