# Supplementary material for: Analysis of bacteria-challenged wild silkmoth, Antheraea mylitta (lepidoptera) transcriptome reveals potential immune genes
Source: BMC Genomics. 2006 Jul 21;7:184. doi: 10.1186/1471-2164-7-184 (PMC1559613; doi:10.1186/1471-2164-7-184)
Supplement: Additional data file 1 — includes the results, methodology and schematic representation of Gene Ontology analysis of the ESTs. [file 1471-2164-7-184-S1.pdf]

Additional data file 1: Gene Ontology annotation of *A. mylitta* immune challenged fat body transcriptome

All the ESTs were assigned a biological process, molecular function and cellular component using Gene Ontology (GO) database. The closest annotated homologue in the GO database was used for assigning these categories. The results of the GO annotation are graphically represented in the Figure below. Based on cellular localization results, most of the gene products were found to be localized in cell (45.4%). In the cell, gene products were most abundant in the intracellular region (66%). A significant portion of proteins was found to localize in the extracellular region (7.7%), revealing that these are likely to be secretory proteins. A majority of proteins from the immune transcriptome were assigned to have the 'binding' function (property of binding macro-molecules) and 67% of these were found to be protein binding. Protein-protein interactions form an integral part of various pathways in immune mechanisms. The next abundant molecular function encountered in the transcriptome was catalytic activity (21.5%). Enzymes also play a major role in the immune system as components of signaling pathways, phenol-oxidase cascade, anti-microbial compounds (lysozyme) etc. In case of biological process, majority of the ESTs belonged to the category of physiological processes (36%). The next abundant category was cellular processes (35%), in which cellular physiological process was about 71%. The abundance of the categories determined by GO highlighted the importance of these processes in immune function.

Materials and Methods

Based on the GO annotation of the closely related homologues, ESTs were assigned to a molecular function, biological process and cellular component from the GO

database (Ashburner *et al.,* 2000 *Nat. Genet.,* **25**: 25-29). GO annotation generates a dynamic controlled vocabulary that can be applied to all organisms, even while knowledge of gene and protein roles in cells is still accumulating and changing. To this end, the Seqdblite FASTA sequence flat file was downloaded from the GO database. By running BLAST against Seqdblite, closest homologue was identified. From BLAST output, molecular functions, biological process and cellular localization were parsed by building an in-house GO database in MySQL from the GO-term-database flat file, downloaded from Gene Ontology Database Downloads (http://www.godatabase.org/dev/). The Perl-DBI was used to interface with MySQL, to extract the parent terms of each individual GO term that were obtained by parsing BLAST output. The output was then represented graphically.

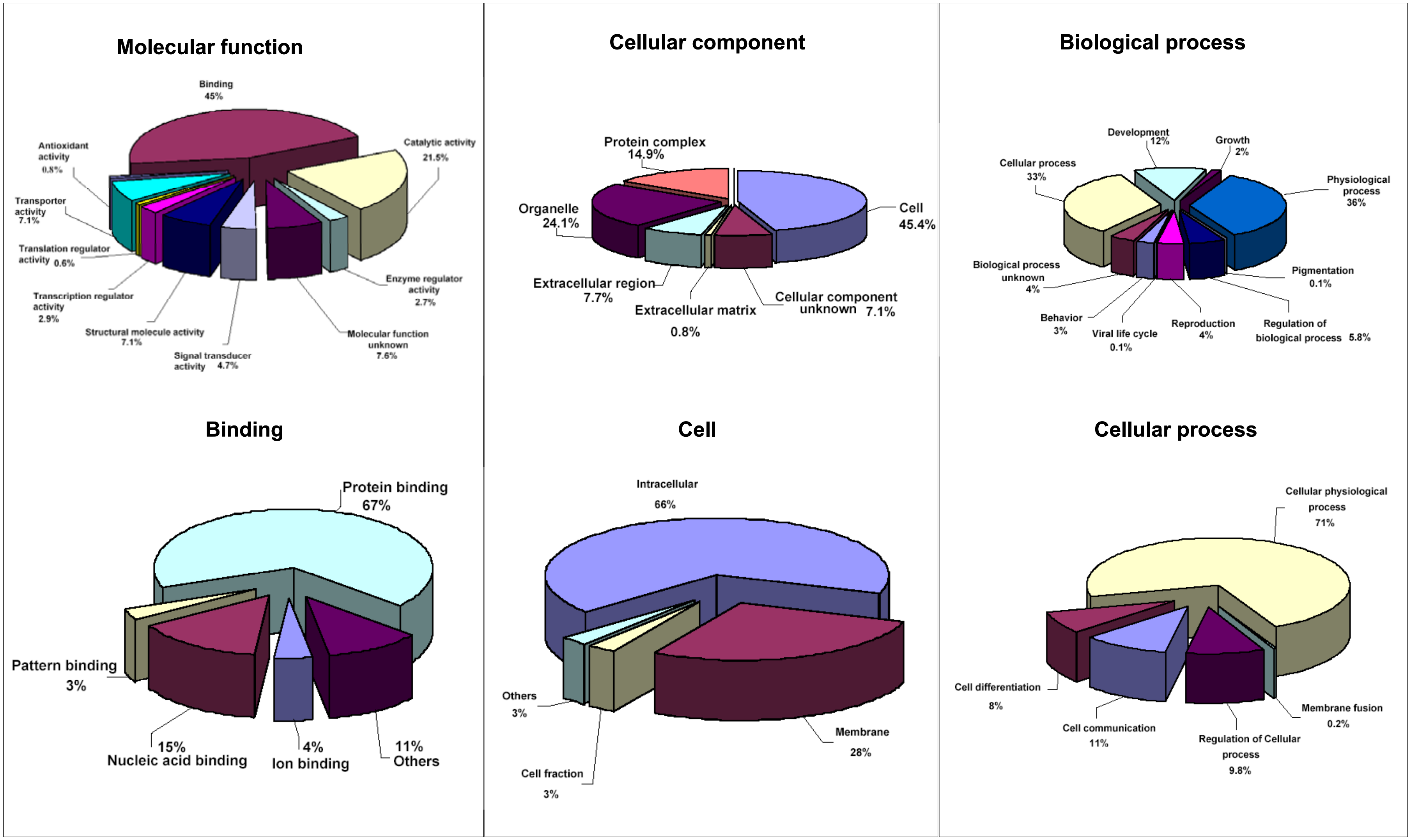

Figure: Annotated GO terms of *A. mylitta* immune challenged fat body EST library. ESTs were classified into different categories based on molecular function, cellular localization and biological process.
