# Supplementary material for: Analysis of bacteria-challenged wild silkmoth, Antheraea mylitta (lepidoptera) transcriptome reveals potential immune genes
Source: BMC Genomics. 2006 Jul 21;7:184. doi: 10.1186/1471-2164-7-184 (PMC1559613; doi:10.1186/1471-2164-7-184)
Supplement: Additional data file 2 — is a table listing the ProDom based annotation of the clusters that did not find any matches in NCBI protein BLAST analysis. [file 1471-2164-7-184-S2.pdf]

Additional data file 2: ProDom based functional annotation of clusters, which had no hits, or showed similarity with unknown/hypothetical proteins in NCBI BLAST.

***- Sequences can be accessed at http://210.212.212.7:9999/PHP/SILKSAT/uniqueseqs/.**

No.	Cluster Name*	ProDom Domains	ProDom Accession Number
1	amfbcontig0019	No hits	
2	amfbcontig0035	No hits	
3	amfbcontig0038	No hits	
4	amfbcontig0043	Ribosome associated membrane RAMP4	PD054374
5	amfbcontig0051	Phosphoribosyltransferase	PD001439
6	amfbcontig0060	CG14984-A	PD306867
7	amfbcontig0061	No hits	
8	amfbcontig0066	Glycoside Hydrolase 29 (Alpha-L-Fucosidase)	PD886685
9	amfbcontig0070	Aminotransferase class-III	PD000493
10	amfbcontig0072, amfb0294	CG33290-PA CG14567 *D. melanogaster*	PD308577
11	amfbcontig0077	CG16926 *D. melanogaster*	PD927175
12	amfbcontig0091	Repressor E1a-stimulated genes	PD152511
13	amfbcontig0092	No hits	
14	amfbcontig0093, amfbcontig0104	Ubiquitin-conjugating enzyme, E2	PD000461
15	amfbcontig0094	Tubulin binding cofactor A	PD010430
16	amfbcontig0095, amfb0124	AICARFT/IMPCHase bienzyme	PD004666
17	amfbcontig0096	No hits	
18	amfbcontig0099	Transmembrane precursor signal TMP21 P23	PD035860
19	amfbcontig0101	Major royal jelly protein	PD014382
20	amfbcontig0108	DEAD/DEAH box helicase	PD000033
21	amfbcontig0111	3-Hydroxyacyl-CoA Dehydrogenase, NAD-binding	PD003524
22	amfbcontig0115	PF08_0013	PD813653
23	amfbcontig0116	Initiation factor subunit	PD444660
24	amfbcontig0121	Actin binding factor	PD129211
25	amfbcontig0122	No hits	
26	amfbcontig0130	Nudix bifunctional Pyrrolidone Carboxyl	PD828714
27	amfbcontig0132	Hydrolase Nitrilase Carbon-Nitrogen Amidohydrolase Family	PD052217
28	amfbcontig0134	L27, Homologue LIN-7 VELI LIN MUS C	PD154569
29	amfbcontig0136, amfb0145, amfb0354, amfb0391	CHCC Zinc –finger domain	PD690711, PD321788

30	amfbcontig0140	Aldehyde cytosolic Dehydrogenase	PDA1A9N1
31	amfbcontig0144	Cysteine twelve *M. sexta*	PD548193
32	amfbcontig0146	Whey acidic protein core region	PD026912
33	amfbcontig0147	Calcium-binding EF-hand	PD000012
34	amfbcontig0149	Kinase Transferase ATP-binding serine/threonine-protein	PD119917
35	amfbcontig0153, amfb1024	RNA-directed RNA Polymerase, P3D protein (polyprotein coat core)	PD686877
36	amfbcontig0155	Synthetase metal-binding	PD606972
37	amfbcontig0156	Purine Phosphorylase 2	PD006911
38	amfbcontig0157	Histone H3	PD000978
39	amfbcontig0158	No hits	
40	amfbcontig0160, amfb1194,	Factor LPS –induced enriched library endosome	PD015270
41	amfbcontig0161, amfb0152, amfb424, afmb0661	ATP dependent RNA Helicase	PD035460, PD035498
42	amfbcontig0162	ADP-specific Phospho Fructokinase/Glucokinase conserved region	PD257594
43	amfbcontig0166, amfb0411	ABC transporter	PD000006
44	amfb0026	No hits	
45	amfb0034	Carbohydrate Kinase, FGGY	PD684127
46	amfb0039	No hits	
47	amfb0047	CG15211-PA RE01453P	PD306644
48	amfb0061	No hits	
49	amfb0065	Synaptobrevin	PD001229
50	amfb0067	GTP-binding elongation factor	PD011419
51	amfb0068	No hits	
52	amfb0081	Peptidase C12, Ubiquitin carboxyl-terminal Hydrolase 1	PD350662
53	amfb0082	Fasciculation elongation Zeta Zygin I Coiled Coil	PD011714
54	amfb0095	WD-40 repeat	PD320067
55	amfb0101	Carboxyl Esterase activity	PD000713
56	amfb0106	Intra cellular protein transport	PD242575
57	amfb0128	No hits	
58	amfb0135	CG7221-PA	PD622051
59	amfb0144	Receptor 20E ecdysone	PD065649
60	amfb0168	Aldo/Keto reductase	PD000288
61	amfb0173, amfb0296	RNA binding ribonuclear protein	PD150747
62	amfb0175	Synapse-Associated Sap47	PD022947
63	amfb0178	No hits	
64	amfb0180	Heme Peroxidase	PD564465

65	amfb0185	No hits	
66	amfb0195, amfb0679	NADH-Ubiquinone (oxidoreductase ubiquinone)	PD072136
67	amfb0197	NAD-dependent Epimerase /Dehydratase	PD291388
68	amfb0199	No hits	
69	amfb0203, amfb1283, amfb0022, amfb1002	RNA-binding region RNP-1 (RNA recognition motif)	PD706470
70	amfb0205	CCR-4 associated factor	PD878878
71	amfb0206	1-(5-Phosphoribosyl)-5-amino-4-imidazole-carboxylate (AIR) Carboxylase	PD002193
72	amfb0213, amfb0426, amfb1367	Ras GTPase	PD000015,
73	amfb0214	Flavoprotein Oxidoreductase	PD115662
74	amfb0221, amfb0879	Beta-3 Karyopherin repeat	PD606523
75	amfb0229, amfb1150	Cytochrome b5	PD000612
76	amfb0233	No hits	
77	amfb0241	Kinase receptor HTK32 Transferase	PD323132
78	amfb0242	Glutamate-cysteine Ligase catalytic subunit	PD235481
79	amfb0244	UPF3 HUPF3A Homolog	PD121682
80	amfb0250	Reticulon	PD186266
81	amfb0263	Short-chain Dehydrogenase/Reductase SDR	PD246641
82	amfb0276	Glutelin	PD192335
83	amfb0278	Chromosome III B0361.6, (protein of unknown function DUF171)	PD111934
84	amfb0280	Salivary MYS2 precursor signal	PD997477
85	amfb0289	Heavy chain inhibitor inter-alpha-trypsin	PD017272
86	amfb0302	CG12489-PA LD18186P	PDA0W1F6
87	amfb0307, amfb0646	ATP-binding MRP homolog nucleotide-binding binding family ATPase chromosome MRP/NBP35 protein	PD092650
88	amfb0311	CG13353-PA	PD315777
89	amfb0312	CG1943-PB CG1943-PA	PD291962
90	amfb0315	DNA Polymerase	PD005357
91	amfb0323	tRNA Pseudouridine Synthase	PD118232
92	amfb0342	Dehydrogenase 3-Hydroxy Acyl Co A	PD466280
93	amfb0349	No hits	
94	amfb0353	ATP epsilon synthase	PD026860
95	amfb0372	Oxidoreductase Flavoprotein FAD Dehydrogenase Acyl-COA	PD000558
96	amfb0374	No hits	

97	amfb0382	No hits	
98	amfb0387	Myb, DNA-binding (nuclear factor transcription MYB-1 box binding)	PD021927
99	amfb0389	No hits	
100	amfb0394	TDET1 MUS ZGC	PD317441
101	amfb0396	WW/Rsp5/WWP	PD354471
102	amfb0397	No hits	
103	amfb0398	EGF-like immunoglobin	PD000227
104	amfb0401	Eukaryotic initiation factor EIF 3E,	PD336869
105	amfb0414	CG14147-PA RE16005P	PD302217
106	amfb0422	Aldose 1 -Epimerase	PD127437
107	amfb0423	Luciferase 4-Monooxygenase	PD870069
108	amfb0430, amfb0108, amfb157	RNA binding region RNP-1	PD743635
109	amfb0433	Epoxide Hydrolase	PD956892
110	amfb0434	EGF-Like Tolloid Metalloprotease	PD719225
111	amfb0464	CG15092-PA	PD491853
112	amfb0481	Sericin	PD018391
113	amfb0490	No hits	
114	amfb0492	General substrate transporter	PD602677
115	amfb0494	Mitochondrial ATP Synthase G subunit	PD015502
116	amfb0501	CG15678-PA SD16010P	PD309707
117	amfb0504	TPR repeat	PD000069
118	amfb0515	Ribosomal transit mitochondrial precursor	PD380986
119	amfb0527	No hits	
120	amfb0533	No hits	
121	amfb0537	Mob1/phocein	PD316458
122	amfb0540	No hits	
123	amfb0544	Perilipin	PD312307
124	amfb0562	Nucleotidyl- Transferase	PD000456
125	amfb0565	No hits	
126	amfb0574	SAICAR Synthetase	PD656412
127	amfb0576	Diacylglycerol Kinase	PD360309
128	amfb0577	Phosphoribosylformylglycinamidine Synthase	PD002258
129	amfb0586	UPF0066 YAEB VIRR uncharacterized enriched product (protein of unknown function)	PD006705
130	amfb0589	No hits	
131	amfb0604	Gamma translocon associated subunit	PD103642
132	amfb0611	RNA binding motif protein 8	PD328190
133	amfb0617	No hits	
134	amfb0618, amfb0824	Farnesoic acid O-methyltransferase activity	PD293778
135	amfb0622	No hits	
136	amfb0625,	Ferredoxin	PD001472

	amfb1187		
137	amfb0638, amfb748	Proteasome component region PCI	PD025198
138	amfb0643	Pyruvate Dehydrogenase	PD186280
139	amfb0649	No hits	
140	amfb0653	Eukaryotic initiation factor EIF 3A	PD014437
141	amfb0655	Peptidylprolyl Isomerase, FKBP-type	PD000420
142	amfb0660	Ribosomal protein S8E	PD122856
145	amfb0665	No hits	
146	amfb0696	Death-associated CG12384-PA DAP-1	PD068445
147	amfb0699	Group XII secretory Phospholipase A2	PD386805
148	amfb0704	No hits	
149	amfb0708	Glycoprotein	PD722012
150	amfb0714	Mnd1	PD110859
151	amfb0722	GTP-CH-I Cyclohydrolase	PD101974
152	amfb0731	Intracellular chloride channel	PD706961
153	amfb0742	No hits	
154	amfb0757	Peptidase, cysteine peptidase active site	PD585084
155	amfb0759	Exonuclease	PD004947
156	amfb0768	CG17233-PA CG17233-PC	PD719922
157	amfb0771	CG11876-PB CG11876-PC	PD807738
158	amfb0777	Acireductone Dioxygenase, ARD	PD006219
159	amfb0778	DCP1-like decapping	PD129069
160	amfb0784	Adenosine Deaminase/editase	PD003961
160	amfb0789	Surface ookinete antigen PFS28	PD008013
161	amfb0801	Phenylalanine-4-Hydroxylase, Amino acid-binding ACT	PD973163
162	amfb0812	No hits	
163	amfb0817	Ubiquitin	PD000119
164	amfb0821	Immunoglobulin-like	PD328728
165	amfb0856	No hits	
166	amfb0866	TMS membrane protein/tumour differentially expressed protein	PD593607
167	amfb0873	Serine-Hydroxymethyl Transferase	PD498026
168	amfb0893	No hits	
169	amfb0895	No hits	
170	amfb0896, amfb0480	Aminotransferase, class V	PD661108
171	amfb0907	No hits	
172	amfb0911, amfb1200	Adenosine/AMP deaminase	PD008716
173	amfb0913	No hits	
174	amfb0916	No hits	
175	amfb0923	Sec61_beta	PD721750

176	amfb0938	No hits	
177	amfb0945	CG33290-PA CG14567-PA	PD308577
178	amfb0953	No hits	
179	amfb0971	Acyl- CoA- binding protein, ACBP	PD351532
180	amfb0975	No hits	
181	amfb0984	Nascent polypeptide-associated complex NAC	PD009422
182	amfb0994	No hits	
183	amfb1000	No hits	
184	amfb1011	No hits	
185	amfb1012	ATP-binding RNA hydrolase	PD410733
186	amfb1014	Calponin repeat muscle actin-binding smooth multigene Calmodulin-binding family homologue	PD105641
187	amfb1028	No hits	
189	amfb1035	FMTB wall peptidoglycan-anchor cell	PD851216
190	amfb1038	Carboxylesterase, type B	PD000605
191	amfb1039	No hits	
192	amfb1052	Mov34/MPN/PAD-1	PD363422
193	amfb1056	No hits	
194	amfb1087	No hits	
195	amfb1093	Tudor domain containing repeat Kinase	PD291106
196	amfb1099	No hits	
197	amfb1123	Lysozyme C Hydrolase	PD000577
198	amfb1134	Heat shock CG6000-PA RH54517P 67B2 CG12279-PA	PD687240
199	amfb1135	TIA-1 homologue	PD978935
200	amfb1149	CG3706-PA SD20854P C05D2.8 EG: BACR7A4.20	PD104297
201	amfb1162	No hits	
202	amfb1167	CG5080-PB LD34147P CG5080-PA	PD324947
203	amfb1170	CG3884-PA CG32633-PA RE21371P ENSANGP00000022546 IB1	PD293778
204	amfb1212	CG6513-PA CG6513-PB	PDA125G1
205	amfb1220	Cys/Met metabolism Pyridoxal-phosphate-dependent enzymes	PD590311
206	amfb1254	No hits	
207	amfb1273	No hits	
208	amfb1276	No hits	
209	amfb1281	No hits	
210	amfb1289	Fibronectin, type III	PD360479
211	amfb1296	No hits	
212	amfb1297	Serine spondin repeat	PD218985
213	amfb1298	No hits	
214	amfb1304	No hits	
215	amfb1308	Sin3 associated polypeptide p18	PD016033
216	amfb1312	Leucine-rich repeat	PD606902

217	amfb1317	No hits	
218	amfb1329	Alpha Amylase, catalytic region	PD479378
219	amfb1331, amfb0488	Nuclear ATP-Binding	PD023569
220	amfb1332	No hits	
221	amfb1334, amfb0475	14-3-3 like Acetylation Phophorylation factor	PD000600
222	amfb1355	Phospholipase/Carboxylesterase	PD352911
223	amfb1356	No hits	
224	amfb1363, amfb0541	Alcohol Dehydrogenase super, zinc-containing	PD002174, PD506568
225	amfb1366	Hydrolase CDS L-Homocystein S-Adenosyl ORFs(Glycosyl transferase 8)	PD125323
226	amfb1370	No hits	
227	amfb1373	Protein of unknown function DUF1319 (ORF1 I polyprotein 12and CDS DNA)	PD087208,
228	amfb1395	Ribosomal peptide MRP-S26	PD423119
229	amfb1402	Integrase	PD107985
