# Supplementary material for: Analysis of bacteria-challenged wild silkmoth, Antheraea mylitta (lepidoptera) transcriptome reveals potential immune genes
Source: BMC Genomics. 2006 Jul 21;7:184. doi: 10.1186/1471-2164-7-184 (PMC1559613; doi:10.1186/1471-2164-7-184)
Supplement: Additional data file 3 — A table representing some of the putative immune genes from the transcriptome is provided as an Additional data file 3 [file 1471-2164-7-184-S3.pdf]

Additional data file 3: A subset of the putative immune genes from the *A. mylitta* transcriptome.

The probable function was assigned based on the homology in NCBI-BLAST.

A.p - *Antheraea pernyi*, S.c.r - *Samia cynthia ricini*, H.c - *Hyalophora cecropia*, H.a - *Helicoverpa armigera*, M.s.- *Manduca sexta*, B.m- *Bombyx mori*, L.o - *Lonomia obliqua*, T.n -*Trichoplusia ni*, P.x.- *Plutella xylostella*, C.s.- *Culicoides sonorensis.*

GenBank accession no.	Nucleotide	E value	Homology	Putative function
DQ666488	977	5e-103	Basic attacin (A.p)	Anti-microbial
DQ666489	984	6e-76	Attacin (S.c.r)	Anti-microbial
DQ666490	429	3e-30	Basic attacin (A.p)	Anti-microbial
DQ666491	924	e-111	Attacin E precursor (H.c)	Anti-microbial
DQ666492	1088	5e-16	Cecropin (H.a)	Anti-microbial
DQ666493	1642	4e-15	Cecropin D (H.c)	Anti-microbial
DQ666494	429	2e-16	Cecropin D (H.c)	Anti-microbial
DQ666495	593	1e-66	Gloverin (M.s)	Anti-microbial
DQ666496	529	8e-54	Gloverin (M.s)	Anti-microbial
DQ666497	1425	0.0	Hemolin (A.p)	Recognition protein
DQ666498	663	2e-88	Hemolin precursor (H.c)	Recognition protein
DQ666499	1145	2e-54	Lebocin (S.c.r)	Anti-microbial
DQ666500	594	8e-11	Lebocin 4 precursor (B.m)	Anti-microbial

DQ666501	713	6e-63	Hypothetical protein (S.c.r)	Anti-microbial
DQ666502	539	4e-50	Hypothetical protein (S.c.r)	Anti-microbial
DQ666503	522	4e-04	Defense protein 4 (L.o)	Unknown
DQ666504	946	2e-47	Lectin 3 (L.o)	Recognition protein
DQ666505	677	1e-70	Peptidoglycan recognition protein (T.n)	Recognition protein
DQ666506	671	2e-82	Peptidoglycan recognition protein 1A (M.s)	Recognition protein
DQ666507	942	2e-66	Serpin 2 (L.o)	Serine protease inhibitor
DQ666508	399	8e-21	Serpin 2 (L.o)	Serine protease inhibitor
DQ666509	607	1e-70	Serpin 2 (L.o)	Serine protease inhibitor
DQ666510	403	4e-40	Serine protease inhibitor 4 (L.o)	Serine protease inhibitor
DQ666511	461	1e-40	Serpin 3 (L.o)	Serine protease inhibitor
DQ666512	301	9e-11	Serpin 2 (B.m)	Serine protease inhibitor
DQ666513	668	1e-35	Serpin 1 (P.x)	Serine protease inhibitor
DQ666514	556	2e-77	Serpin 6 (M.s))	Serine protease inhibitor
DQ666515	448	6e-16	Protease inhibitor 1 (L.o)	Protease inhibitor
DQ666516	377	5e-12	Protease inhibitor 3 (L.o)	Protease inhibitor
DQ666517	485	6e-16	Protease inhibitor 1 (L.o)	Protease inhibitor
DQ666518	584	2e-17	Protease inhibitor 1 (L.o)	Protease inhibitor

DQ666519	615	5e-06	Protease inhibitor 1 (L.o)	Protease inhibitor
DQ666520	579	2e-28	Protease inhibitor 6 (L.o)	Protease inhibitor
DQ666521	569	2e-28	Protease inhibitor 6 (L.o)	Protease inhibitor
DQ666522	592	3e-07	Thiol protease-like (C.s)	Protease inhibitor
DQ666523	400	3e-08	Silk proteinase inhibitor (B.m)	Protease inhibitor
DQ666524	548	1e-36	Kazal-type proteinase inhibitor (M.s)	Protease inhibitor
DQ666525	940	4e-04	Seroin 2 (B.m)	Antimicrobial
