# Supplementary material for: Analysis of bacteria-challenged wild silkmoth, Antheraea mylitta (lepidoptera) transcriptome reveals potential immune genes
Source: BMC Genomics. 2006 Jul 21;7:184. doi: 10.1186/1471-2164-7-184 (PMC1559613; doi:10.1186/1471-2164-7-184)
Supplement: Additional data file 4 — is a table that lists the hits obtained by NCBI protein BLAST analysis of DFP-1, 2, 3 and 4. [file 1471-2164-7-184-S4.pdf]

Additional data file 4: Proteins similar to DFPs as determined by protein BLAST in NCBI. The *B. mori* proteins similar to DFPs were obtained by blastp analysis at the website-http://silkworm.genomics.org.cn/. The corresponding gene number has been mentioned. The hits were grouped into 3 categories based on their origin - lepidopteran insects, insects other than Lepidoptera and vertebrates

PROTE -IN	LEPIDOPTERA			OTHER INSECTS			VERTEBRATES		
	Organism	Function	Accession No.	Name	Function	Accession No.	Name	Function	Accession No.
DFP-1, DFP-3	*B. mori*<br>*Samia cynthia ricini*<br>*L. obliqua*	Unknown	Bmb041147<br>BAD05929<br>AAV91350	*A. gambiae*<br>*D. melanogaster*	Unknown	ENSANGP0000000 354<br>CG8399-PA	*Pan troglodytes*<br>*Mus musculus*	Predicted- stromal cell receptor factor - 2	XP_517470<br>AAH27770
DFP-2	*B. mori*<br>Rest same as DFP-1 and 3.	Unknown	Bmb024557	Same as DFP-1 and 3	Same as DFP-1 and 3	Same as DFP-1 and 3	Same as DFP-1 and 3	Same as DFP-1 and 3	Same as DFP-1 and 3
DFP-4	*L. obliqua*<br>*B. mori*<br>*H. armigera*	Cecropin	AAV91462<br>BAE53371<br>AAX51193	None	-	-	No significant similarity	-	-
