# Supplementary material for: Analysis of bacteria-challenged wild silkmoth, Antheraea mylitta (lepidoptera) transcriptome reveals potential immune genes
Source: BMC Genomics. 2006 Jul 21;7:184. doi: 10.1186/1471-2164-7-184 (PMC1559613; doi:10.1186/1471-2164-7-184)
Supplement: Additional file 5 — Primer sequences of the 15 transcripts analysed by RT-PCR have been provided in Additional data file 5. [file 1471-2164-7-184-S5.pdf]

Additional data file 5: Primer sequences used for RT-PCR profile. * GenBank accession numbers are not available for these sequences, which can be accessed at http://210.212.212.7:9999/PHP/SILKSAT/uniqueseqs/.

**Gene**	**GenBank accession number**	**Forward primer (5′-3′)**	**Reverse primer** (5′-3′)
Attacin1	DQ666490	TTCCACCCTGCGTTAAAGTC	ACAGAATCGGTGCATCAGC
*Attacin 2**	amfb1278	CGGCGAGCTGGTAAAGTATC	CAGAACCGATTGCGCTTAAC
Cecropin1	DQ666492	TCGATGCCTACAGTTTCGAG	CTTCCTGTTCGTTTGCCTTC
Cecropin 2	DQ666493	CTTCCCTTTAGCCAATGCTG	TGTTGTTCGTATTCGCTTGC
DFP-1	DQ666501	TTGTCCGCTGACTCAGAATG	TACAATCAGCGGCAAGACAC
DFP-4	DQ666503	TCGTTCGACAGAACTTGAAGG	TCAGATTTGTTTGCCGTCTG
Gloverin	DQ666495	TGCCTCCTGGATACGAAAAG	TTACCGAATAACCCGTCGTC
Hemolin	DQ666498	CTGACGGAAACCTCAGCTTC	TGGCACTTCTTCATCGACAG
Lebocin 1	DQ666499	AACAGCCATAACGGTTCGTC	TTGGTCGTCGATACGACTTG
Lebocin 2	DQ666500	GAAGGCAAGCTTGAGTGGTC	CCTCAGTGTTGTTCGTGCAG
Lectin	DQ666504	CGTGCGACGATGTATTTGAG	GTCCGAACCGATGAAACTTG
PGRP-1	DQ666506	ACGAAGGATCTGGTTGGTTG	ACGCCGCATCTAAGTAAAGC
*PGRP-2**	amfbc0145	AACCTCAACGTGACCAGGAC	GCAGCACACGTGAGCAATAC
Seroin	DQ666525	TGATGTTCCCTATGCCACAC	ACCGGAGAAATGTTCGTTTG
*Serpin**	amfb0553	TGACTTTGGGTGCAACTGAC	ATGCATATGGTCCCTCGTTC
Proteinase inhibitor	DQ666519	GACCGTTAATGGCTGCAATC	CTTGCCGGTATCATTTCCAG
Actin		GGCATGGGACAGAAGGACT	TAGTGACGATTCCGTGTTCG
